# A Miniature Bio-Photonics Companion Diagnostics Platform for Reliable Cancer Treatment Monitoring in Blood Fluids

**DOI:** 10.3390/s21062230

**Published:** 2021-03-23

**Authors:** Marianneza Chatzipetrou, Lefteris Gounaridis, George Tsekenis, Maria Dimadi, Rachel Vestering-Stenger, Erik F. Schreuder, Anke Trilling, Geert Besselink, Luc Scheres, Adriaan van der Meer, Ernst Lindhout, Rene G. Heideman, Henk Leeuwis, Siegfried Graf, Tormod Volden, Michael Ningler, Christos Kouloumentas, Claudia Strehle, Vincent Revol, Apostolos Klinakis, Hercules Avramopoulos, Ioanna Zergioti

**Affiliations:** 1 Institute of Communication and Computer Systems (ICCS), National Technical University of Athens, Heroon Polytehneiou 9, 15780 Athens, Greece; mchatzip@mail.ntua.gr (M.C.); lgou@mail.ntua.gr (L.G.); m.v.dimadi@gmail.com (M.D.); ckou@mail.ntua.gr (C.K.); hav@mail.ntua.gr (H.A.); 2 Biomedical Research Foundation of the Academy of Athens, Soranou Ephessiou 4, 11527 Athens, Greece; gtsekenis@bioacademy.gr (G.T.); aklinakis@bioacademy.gr (A.K.); 3 Future Diagnostic Solutions B.V., Nieuweweg 279, 6603 BN Wijchen, The Netherlands; Vestering-Stenger.R@future-diagnostics.nl (R.V.-S.); Lindhout.E@future-diagnostics.nl (E.L.); 4 LioniX International B.V., P.O. Box 456, 7500 AL Enschede, The Netherlands; f.schreuder@lionix-int.com (E.F.S.); g.a.j.besselink@lionix-int.com (G.B.); r.g.heideman@lionix-int.com (R.G.H.); h.leeuwis@lionix-int.com (H.L.); 5 SurfiX B.V., Plus Ultra Building—Bronland 12 B-1, 6708 WH Wageningen, The Netherlands; anke.trilling@surfix.nl (A.T.); luc.scheres@surfix.nl (L.S.); Adriaan.vanderMeer@surfix.nl (A.v.d.M.); 6 CSEM SA, Untere Gründlistrasse 1, 6055 Alpnach, Switzerland; Siegfried.GRAF@csem.ch (S.G.); tormod.volden@csem.ch (T.V.); vincent.revol@csem.ch (V.R.); 7 LRE Medical, Hofer Strasse 5, 86720 Noerdlingen, Germany; Ningler@lre.de (M.N.); strehle@lre.de (C.S.)

**Keywords:** analysis of blood serum, cancer therapy treatment monitoring, point of care, biosensor, optical sensor

## Abstract

In this paper, we present the development of a photonic biosensor device for cancer treatment monitoring as a complementary diagnostics tool. The proposed device combines multidisciplinary concepts from the photonic, nano-biochemical, micro-fluidic and reader/packaging platforms aiming to overcome limitations related to detection reliability, sensitivity, specificity, compactness and cost issues. The photonic sensor is based on an array of six asymmetric Mach Zender Interferometer (aMZI) waveguides on silicon nitride substrates and the sensing is performed by measuring the phase shift of the output signal, caused by the binding of the analyte on the functionalized aMZI surface. According to the morphological design of the waveguides, an improved sensitivity is achieved in comparison to the current technologies (<5000 nm/RIU). This platform is combined with a novel biofunctionalization methodology that involves material-selective surface chemistries and the high-resolution laser printing of biomaterials resulting in the development of an integrated photonics biosensor device that employs disposable microfluidics cartridges. The device is tested with cancer patient blood serum samples. The detection of periostin (POSTN) and transforming growth factor beta-induced protein (TGFBI), two circulating biomarkers overexpressed by cancer stem cells, is achieved in cancer patient serum with the use of the device.

## 1. Introduction

In the medical diagnostics industry, there is an increasing need for robust, reliable, accurate and fast devices for early diagnosis, patient screening and disease monitoring, which will also facilitate the materialization of the concept of Personalized Medicine [1,2]. There is a special interest in cancer, as it is among the leading causes of death worldwide, accounting for 14.1 million new cancer cases and 8.2 million cancer deaths per year (13% of all deaths worldwide) [3], of which more than 90% are attributed to tumor metastasis [4]. In the EU, cancer-related mortality surpassed that from cardiovascular disorders representing the leading cause of death (over 25%), while, in the years to come, cancer incidents and deaths are expected to rise in line with the increase of life expectancy. Nevertheless, according to the World Health Organization (WHO), death rates due to cancer can be curbed and patients can have a higher chance of getting cured if cancer is diagnosed early and is treated adequately through personalized therapeutic schemes.

Currently, the vast majority of cancer diagnostics rely on are polymerase chain reaction (PCR), enzyme linked immunosorbent assay (ELISA), radioimmunoassay (RIA), immunohistochemistry (IHC) and flow cytometry [5,6,7], which are based on genomic and proteomic molecular analyses. Even though these techniques are proven efficient, they are performed in hospitals or laboratories that are often located far from the actual site of patient care and need to be equipped with large and complex instruments often requiring highly skilled personnel to operate. A major challenge, therefore, in cancer therapeutic monitoring is the development of robust, reliable and even portable diagnostic devices that will allow the detection of cancer biomarkers in locations such as community hospitals, the doctor’s office or perhaps even at home in the future.

The clinical importance of cancer stem cells and their role in tumor growth, metastasis, therapeutic resistance and tumor recurrence is unequivocal. Cancer stem cells represent the most aggressive/tumorigenic cell compartment within tumors and are known to support primary tumor growth and to migrate to distant tissues and establish secondary tumors (metastases). They are also believed to survive chemotherapy and upon completion of treatment, they grow back leading to tumor recurrence. These recurrences evolve to ever more resistant tumors with fatal outcome. Thus, developing diagnostic tools that allow for the detection of cancer stem cells represent an important step in the field. The simultaneous detection of two independent proteins, each recognized by specific biorecognition elements, substantially increases the specificity, reliability and the accuracy of early cancer diagnosis as well as treatment monitoring.

Biosensor devices for cancer detection are necessary for the development of such integrated devices and depending on the target, may use antibodies, nucleic acids or other biorecognition elements [8]. As a transducer, depending on the biological response, several types have been used, such as electrochemical [9], optical [10] and mass-based transducers [11]. Electrochemical transducers are the ones most commonly used in PoC devices as they have very low cost, are simple to use and can be miniaturized and mass produced [12,13]. Furthermore, optical transducers advance over the most categories as they are capable of detecting multiple cancer biomarkers [14] and can have improved sensitivity with different geometries of the waveguides.

In this paper, we present a miniaturized, ultra-sensitive and reliable Point-of-Care (PoC) device with a disposable microfluidic cartridge for the monitoring of cancer biomarkers in blood. The novelty of the photonic biosensor device is based on the combination of several techniques from multiple disciplines in order to fully integrate the aMZI sensor with light sources and detectors into a disposable cartridge. The developed platform can be used with the simultaneous measurement of up to six different biomarkers. However, we focused on the detection of the periostin POSTN and transforming growth factor beta-induced protein TGFBI proteins, which, in cancer cells, are regulated by the stem cell transcription factor Snail (SNAI1).

## 2. Materials and Methods

### 2.1. Integrated Optical Sensing Chips

The photonic platform relies on the use of Si_3_N_4_ waveguides (TriPleX) technology to make an array of asymmetric Mach–Zehnder Interferometers (aMZIs), which are used as highly sensitive refractive index based photonic sensors for the analysis of blood circulating biomarkers, and are described in detail elsewhere [15,16].

The design of the waveguide used for the sensor design is based on an asymmetric double stripe Triplex geometry [17], which is specifically optimized for a wavelength of 850 nm. The waveguide structure is built with two Si_3_N_4_ layers and an intermediate SiO_2_ layer. It contains a thin 35 nm stoichiometric Si_3_N_4_ bottom stripe, a 100 nm intermediate layer of LPCVD SiO_2_ (TEOS) and a top stripe of 78 nm stoichiometric Si_3_N_4_. These three layers together form the core of the waveguide and are built upon a Si wafer with 6-micron thermal oxide. On top the waveguide structure, a top cladding of a LPCVD SiO_2_ (TEOS) layer is deposited to completely encapsulate the waveguide with SiO_2_.

**Figure 1:**
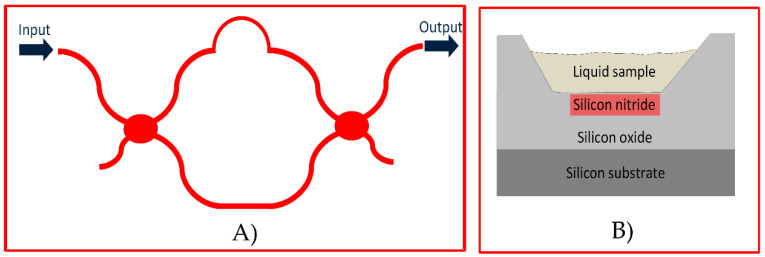
(**A**) Layout of a typical asymmetric Mach Zender Interferometer (aMZI); (**B**) waveguiding structure of the aMZI in this sensor based on the TriPleX platform.

Figure 1A presents the layout of an aMZI that serves as the interferometer in our sensor. When light comes from the input port, it is coupled to the waveguide of the aMZI and finds its way out of it through the output port of the element. The transmission of optical power at the output port has a transfer function, which is described by Equation (1):

(1) $$T(\varphi )=\frac{1}{2}\cdot (1+\cos\Delta \varphi )$$

The same equation can be also expressed as a function of the operating wavelength *λ* (Equation (2)):

(2) $$T(\lambda )=\frac{1}{2}\cdot (1+\cos(\frac{2\pi }{\lambda }\cdot L\cdot \Delta n_{eff}))$$

where *n_eff_* is the effective index of the propagating mode and *L* the physical length of the aMZI sensing arm. When *φ* is a multiple of 2*π*, the interference is constructive.

The free spectral range (FSR) of the aMZI that describes the spectral distance between successive interferences near a wavelength *λ*_0_ is given by (3):

(3) $$FSR=\frac{\lambda_{0}^{2}}{n_{g}\cdot L}$$

In this relation, *n_g_* is the group index of the propagating mode that is associated in turn with the effective index and the derivative of the effective index at *λ*_0_ is as follows (Equation (4)):

(4) $$n_{g}=n_{eff}-\lambda_{0}\frac{dn_{eff}}{d\lambda }$$

Figure 1Β presents the cross-section of the waveguiding structure of the aMZIs in this sensor. It is based on the TriPleX platform, which combines a thin lower and an upper silicon nitride strip as the waveguide core and surrounding silicon oxide layers as the cladding. In the location of the aMZIs’ sensing arms on the optical chip, the upper silicon oxide layer is removed, making the surface of the strip accessible to the liquid biochemical samples. Depending on the application and the employed biochemical technique, the target molecules can get close to this surface or be truly captured on this surface resulting in either case in a local change of the refractive index in the upper cladding of the aMZI sensing arm. This change is sensed by the evanescent field of the propagating mode and modifies its effective index. This modification can then be quantified through the measurement of the aMZI wavelength shift.

The sensitivity of the aMZI directly depends on the arm length and the FSR of the photonic structure. Equation (5) provides the sensitivity as:

(5) $$Sensitivity=\frac{\Delta \lambda }{\Delta n_{eff}}=\frac{L\cdot FSR}{\lambda_{res}}$$

where *L* is the length of each arm of the aMZI and *λ_res_* the resonance operating wavelength. There are two ways to increase the sensitivity. One way is by choosing larger *FSR* by implementing a shorter pathlength difference between the sensing and the reference arm (decrease the asymmetry) or/and increasing the pathlength of each arm. In the BIOCDx sensor, an aMZI with sensitivity up to 5000 nm/RIU is used, which corresponds to an FSR equal to 1500 pm and sensing arm length of 12.5 mm.

The main criterion for the performance quantification of the aMZI sensors is the limit of detection (LoD), which is defined as the minimum refractive index change that can be reliably detected by the system in the upper cladding of the aMZI. Depending on the size of the target molecules and the way in which they are captured near the surface of the aMZI, the LoD can be translated into the minimum quantity of the molecules that can be detected and quantified. As mentioned, the LoD is associated with the resolution R and the sensitivity *S* of the system through the relation (Equation (6)):

(6) $$LoD=\frac{R}{S}$$

where *R* is the resolution and *S* the sensitivity of the system. The resolution can be established as the 3*σ* standard deviation of the measured resonance position 3*σ* (*meas*-*n*), which represents the total measurement noise (Equation (7)):

(7) $$3\sigma_{meas-n}=3\cdot \sqrt{\sigma_{ampl-n}^{2}+\sigma_{spect-n}^{2}+\sigma_{temp-n}^{2}}$$

In this equation *σ*_(*ampl-n*)_, *σ*_(*spect-n*)_ and *σ*_(*temp-n*)_ represent the individual contributions to the measurement noise due to the amplitude, spectral and temperature noise, respectively. In our case, the 3*σ*_(*meas-n*)_ is measured 0.8 pm. These parameters lead to a LoD of 16 × 10^−8^ RIU.

**Figure 2:**
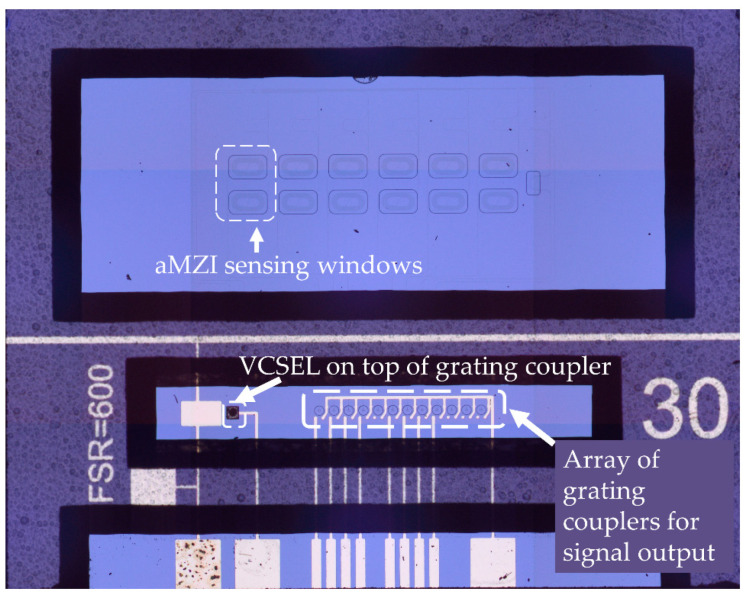
aMZI chip with hybrid integration of vertical-cavity surface-emitting laser (VCSEL) and Photodiodes.

Briefly, the photonic chip, illustrated in Figure 2, consists of 1 input channel split on-chip into 8 individual output channels. Each channel excites one aMZI sensor element, of which 6 are used to detect the biological reaction where the arm length of the interaction window and the free spectral range, which is related to the asymmetry between the two arms, is tuned to enhance the sensitivity. One aMZI is used for low sensitivity applications and is used to highlight the bulk refractive index changes and the last one is used for internal reference and calibration purposes. In order to detect the biological reaction, the 6 individual sensor elements have to be chemically modified for the binding of the biomaterials and subsequently biomodified with the chosen antibodies that will induce the biological reaction. Each aMZI sensor is composed out of a signal arm and a reference arm, with a sensing window and a reference window, respectively. This balanced design of the sensor allows the detection and direct compensation for bulk and background effects for 6 analytes simultaneously. The detection of the biomarker is observed by measuring the wavelength shift of the sinusoidal aMZI transfer function, which is directly related to the phase change of the aMZI, and which is then processed in an accurate and efficient manner with Fourier analysis as described in [18,19]. The photonic chips are designed for single-mode operation at 850 nm and accommodate the integration of a VCSEL (vertical-cavity surface-emitting laser) as a small tunable light source and a photodiode array for measuring the aMZI response signals, providing true potential for sensor compactness and miniaturization.

### 2.2. Bio-Modification of the Photonic Chips

Two complementary biofunctionalization approaches were developed for the photonics biosensor device. The first concerns the material-selective COOH/PEG (Carboxylic acid/Polyethylene glycol) protocol, which has been described in detail elsewhere [20]. Briefly, the Si_3_N_4_ waveguide is coated with a COOH-terminated layer (which can be used to covalently immobilize the antibodies) and the surrounding SiO_2_ is coated with a protein-repellent PEG layer. After activation of the COOH coating via NHS/EDC chemistry, the chips where biomodified by spotting (by a commercial printer) of the anti-POSTN antibody (Stiny-1 clone), the anti-TGFBI antibody (348506 clone) and an antibody used as a negative control (mouse IgG) on the different aMZIs sensors of one chip. The alternative biomodification approach involved a material-selective chemistry based on the treatment of silicon nitride with hydrofluoric acid followed by glutaraldehyde (further referred to as HF chemistry) [21]. Upon chemical functionalization of the surfaces, the same antibodies previously described were deposited onto the aMZIs with the use of the Laser Induced Forward Transfer (LIFT) technique. The LIFT setup consists of a pulsed Nd:YAG laser (λ = 355 nm, 10 ns pulse duration) and a high-power imaging micromachining system and is described in detail elsewhere [22]. Briefly, the laser beam is directed through the optical setup to a donor substrate with an absorbing interlayer and the liquid to be deposited. As the laser beam irradiates the donor substrate, a liquid droplet is created and, thus, deposited to the sensor substrate, which is placed parallel in close proximity to the donor substrate. The chips were subsequently incubated either with spiked samples that contained increasing concentrations of TGFBI followed by samples that contained increasing amounts of POSTN, or in the case of the chips treated with the HF-chemistry, with cancer patient samples. In both cases, the antibodies were incubated onto the functionalized surfaces for half an hour and at a final concentration equal to 100 μg/mL in PBS.

**Figure 3:**
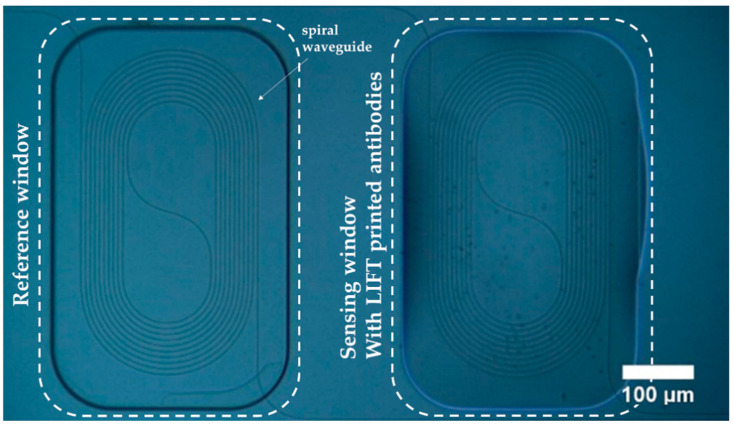
Optical microscopy image of the Laser Induced Forward Transfer (LIFT) printed biomaterials on the sensing aMZI of the photonic chip.

Figure 3 depicts an optical microscopy image of a pair of the aMZIs that exist in a balanced photonic chip. The left arm corresponds to the reference aMZI arm. This is a chemically modified spiral waveguide with a pathlength of 12.5 mm. The reference window of this arm is open and, thus, the waveguide is exposed to the same analyte as the sensing arm. The right arm corresponds to the sensing aMZI. This arm has been chemically modified and has an open sensing window to detect the analyte as well. Furthermore, the sensing window is filled with the biomaterial solution (containing the anti-POSTN or anti-TGFBI antibody), which has been LIFT spotted. As a result, the sensing arm is expected to specifically bind the analyte in the sample solution.

### 2.3. Microfluidic Cartridge

**Figure 4:**
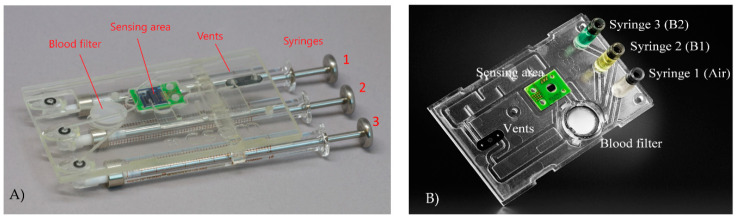
(**A**) Picture of the microfluidic cartridge used during the validation process. Syringe 1 (air) is used to drive the patient sample through the blood filter. Syringe 2 (**B1**) contains priming/washing buffer. Syringe 3 (**B2**) contains a second buffer specific to the application. Light sensors are used to detect the arrival of liquid fronts. Two waste chambers collect the liquids used in the experiment, and only air escapes through the vents. An alternating valve actuation on the vents steers the flow through the sensor area or a bypass channel, respectively. (**B**) Picture of the final injection molded cartridge revision also shown inserted in the instrument in Figure 5.

The microfluidic cartridge, illustrated in Figure 4A,B, is used as a pre-analytical preparation step for whole blood so as to extract plasma and serum from blood while retaining the proteins, and to multiplex the flow of the fluids on the photonic sensor. The separation of red blood cells (RBCs) from plasma and serum is achieved by a Vivid GX plasma separation membrane. A blocking agent PBS-Tween with 5% BSA (Bovine Serum Albumin) is applied by dip-coating for 1 h with subsequent drying in a vacuum chamber for 48 h at 50 mbar to minimize non-specific binding in the cartridge. Fluid actuation is achieved through positive pressure applied on the liquid by on-chip syringes, in order to avoid outgassing and to minimize the influence of air bubbles. Reflective photo-sensors are used to detect air bubbles and direct them to the bypass waste. To guide the flow, an array of 3 syringes together with 2 valves are used. This design allows us to have a simple mechanical interface on the syringe side and a simple plunger on elastomer combination on the waste vents. In a last step, the cartridge shown in Figure 4A, which was used to perform all validation tests, has been further optimized for injection molding and is shown in Figure 4B.

### 2.4. Optical Measurement Experimental Setups

Aiming towards the development of an integrated photonic biosensor device, there is a need to evaluate each platform that consists part of the device prior the integration. To evaluate the performance of the photonic biosensors, two different optical setups have been developed. One consists of a breadboard system with an integrated circuit for the coupling of light. This circuit consists of a fiber array unit with one polarization maintaining (PM) fiber and 7 single-mode fibers at 850 nm. As light source, a single-mode VCSEL with transverse electric (TE) polarization is used. The VCSEL is biased above its lasing threshold, and driven by a sawtooth injection current waveform, with operating temperature at 30 °C by using active cooling. The optical signals from the aMZI output waveguides are guided to an 8-fold array of Si photodiodes, and a low noise transimpedance amplifier (TIA) array with adjustable gains is used at the back end in order to amplify the generated photocurrents and adjust their levels within the operating range of the data acquisition system. The samples are transferred to the sensing window of the chip via a microfluidic system. This system comprises a set of port valves, a peristaltic pump and tubing that interconnects the individual elements and ends at the input port of a chip holder. During the measurements, the flow rate of the peristaltic pump is kept constant at 1 microliter per second (µL/s). The data acquisition system and the microfluidic system are controlled via a software platform (LabVIEW). The FFT-based processing algorithm has been incorporated into this platform in order to perform the signal processing in real-time using the same software environment.

### 2.5. Integrated Photonic Biosensor Device for Cancer Treatment Monitoring

**Figure 5:**
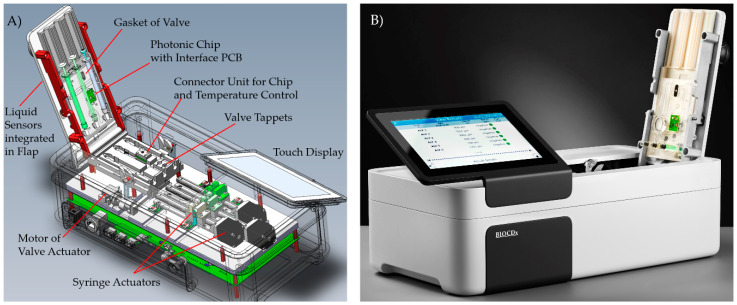
BIOCDx photonic biosensor device with inserted cartridge, (**A**) 3D model, and (**B**) final instrument.

The integrated photonic biosensor device (Figure 5) allows for processing of the micro-fluidic cartridge by controlling the fluidics and driving the photonic chip. Stepper motors allow a continuous flow of the liquid from a selected syringe in the range of 4 to more than 200 µL/min. Five reflective photo-sensors can detect transitions between air and liquid at certain positions in the liquid channels of the cartridge. The valve actuator is realized by a servo motor for pressing spring-loaded tappets to a rubber gasket. The PoC device establishes electrical contact to the photonic chip integrated in the cartridge. A heating resistor on the backside of the chip and an infra-red temperature sensor in the instrument keep the chip temperature on a constant level. A sawtooth-shaped current with a frequency of 10 Hz drives the VCSEL. Signals from the eight photo-diodes on the photonic chip are read out in parallel with a rate of 1000 samples per VCSEL current cycle, i.e., 10 kHz per photo-diode. The sine-like interference patterns from the eight photo-diodes are analyzed with a Fourier-based algorithm. This allows tracking the phase shifts caused by refractive index changes due to the analyte concentration in the sample.

All hardware components, as presented in Figure 5A, were integrated into a compact measurement system. For experimental use, this setup can be used via a PC tool allowing flexible configuration of measurement sequences. Additionally, a complete instrument was created controlled by the operator via a touch display.

## 3. Results and Discussion

**Figure 6:**
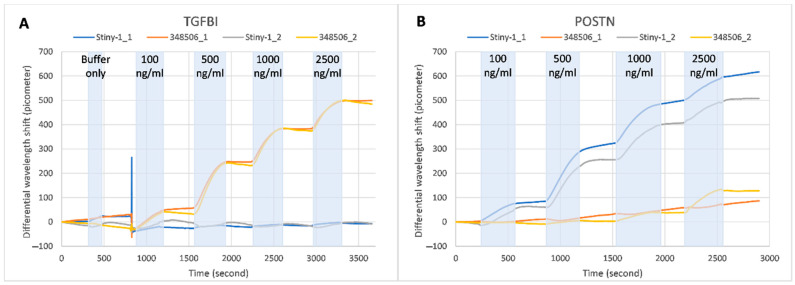
Sensorgram of the multiplex binding of recombinant TGFBI (**A**) or recombinant POSTN (**B**) during incubation with spiked buffer sample employing differently modified (348506 or Stiny-1 and mouse IgG as negative control) aMZI sensors. The differential wavelength shift refers to the signal on the antibody spotted aMZI minus a specific signal on the mouse IgG modified aMZI. Those results are recorded with the optical measurement experimental setup.

The antibody-functionalized biosensor was initially tested with various concentrations of both recombinant POSTN and TGFBI, on the same chip, using water solutions (Tris-0.05% Tween20-1x non-fat dry milk) spiked with increasing concentrations of TGFBI (from 100 ng/mL to 2500 ng/mL) and POSTN (from 100 ng/mL to 2500 ng/mL). Towards this aim, two out of the six sensing aMZIs were biomodified with the anti-TGFBI antibody (clone 348506), two with the anti-POSTN antibody (clone Stiny-1) and the remaining two with polyclonal mouse IgG, which was used as a negative control. Figure 6 depicts the differential signals recorded following incubation of the sensor with the samples described above. The differential signals were obtained by correcting the signal of each of the monoclonal antibody-modified sensors with its nearest reference mouse IgG-modified sensor. The results show a significant amount of specific binding, while the multiplex detection of different analytes is also demonstrated as the binding of both TGFBI and POSTN samples to their immobilized antibodies can take place on the same photonic chip. Binding was fully specific in the case of TGFBI even at the highest applied concentration of 2.5 µg/mL (Figure 6A), while in the case of POSTN, some degree of non-specific binding was found at POSTN concentrations of 1.0 µg/mL and higher (Figure 6B). The reason for the last-mentioned cross-reactivity is not known. Regarding the assay sensitivity, the functionalized aMZIs with the anti-POSTN antibodies successfully detected the samples with concentration as low as 10 ng/mL (results not shown), and detection of even lower amounts seemed to be very feasible. Comparable results were obtained with functionalized aMZIs with anti-TGFBI antibodies.

Subsequently, to validate the developed platform with real patient samples, a biomodified photonic sensor was incubated with 10% serum from patients diluted in the same binding buffer described earlier. The photonic sensor was functionalized with the material-selective chemistry (HF treatment), while the antibodies against POSTN and TGFBI were immobilized onto different sensing aMZIs with the use of the LIFT technique. Two of the aMZIs were not functionalized with antibodies as an additional negative control.

**Figure 7:**
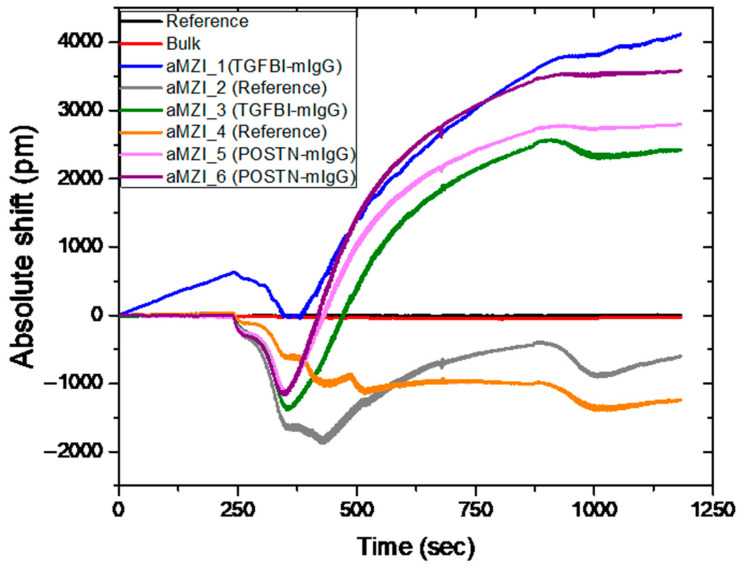
Detection of recombinant POSTN and TGFBI in 10% patient serum sample. These results are recorded with the optical measurement experimental setup.

Figure 7 depicts the absolute wavelength shift over time for the detection of recombinant POSTN and TGFBI in 10% breast cancer patient serum sample with the BIOCDx photonic sensor. Subsequent washing with water and buffer with BSA is performed before the exposure of the sensor to the real patient sample. The red and black lines correspond to the bulk and reference aMZIs, which are used for calibration and for the detection of any bulk refractive index effect (such as temperature variations). The fact that we do not observe a signal from these aMZIs is an indication that the photonic chips are functional. aMZIs 1 and 3 (blue and green) correspond to the interferometers modified with anti TGFBI antibody. In those interferometers, the sensing arm is LIFT spotted with anti TGFBI antibody, while the reference arm is LIFT spotted with polyclonal mIgG. Both arms are exposed to the same sample simultaneously and the reference arm acts as a correction for bulk and background effects to the recorded wavelength shift, as the absolute signal of each aMZI contains the information from both arms; thus, it takes into account all the background effects and discards the biochemical noise. aMZIs 5 and 6 (pink and magenta) correspond to the interferometers modified with anti POSTN antibody. Accordingly, in those interferometers, the sensing arm is LIFT spotted with an anti POSTN antibody while the reference arm is LIFT spotted with polyclonal mIgG. aMZIs 2 and 4 (grey and orange) are not LIFT spotted with any antibodies and are used as a negative control upon incubation in the 10% serum sample. Those aMZIs are blocked with BSA (1% *w/v*) prior the incubation of the sensor with the 10% serum sample. Looking at the aMZIs that are not spotted, an opposite shift is observed for the signal that corresponds to the binding of the biomarkers. This shift was observed in all the tests with real samples and has been used as a baseline to the quantification of the response of the sensors spotted with anti-TGFBI and anti-POSTN. A possible explanation to that could be the dynamic nature of the measurements that depict the changes that occur on the sensor surface as the sample is flowed through. As it has also been observed in SPR measurements (surface plasmon resonance), such shifts could indicate structural rearrangements of the surface or even reversible adsorption kinetics between the sample and sensor surface [23,24]. In our case, those shifts could be due to some form of specific interaction or adsorption of sample being passed above the sensors and the BSA blocked surfaces. The rest of the aMZIs that were functionalized with anti-POSTN or anti-TGFBI were able to detect TGFBI and POSTN in the spiked patient’s serum. The same sample was analyzed with three more biomodified photonic chips that had been functionalized in exactly the same way. The mean shifts recorded for TGFBI were 1700 ± 520 pm, while for POSTN was equal to 2900 ± 900 pm. The variability exhibited by the shifts recorded by aMZIs functionalized with the same antibody could be explained taking into consideration a number of factors that can ultimately add up in the recorded discrepancies. As far as the sensing platform itself is considered, we have observed that despite the consistently reproducible results that we have obtained when the individual components were developed and tested with spiked samples (Figure 6), in the presence of serum samples, we observe higher inconsistences. This could be attributed to antigen depletion phenomena [25], differences in the in the sensitivity of aMZIs on the same chip or in the uniformity of biofuntionalization. Currently we are working both towards improving on sensor fabrication and biofunctionaliation, but also towards gaining a better understanding of diffusion kinetics, flow rates and antigen depletion within the fluidic chamber.

**Figure 8:**
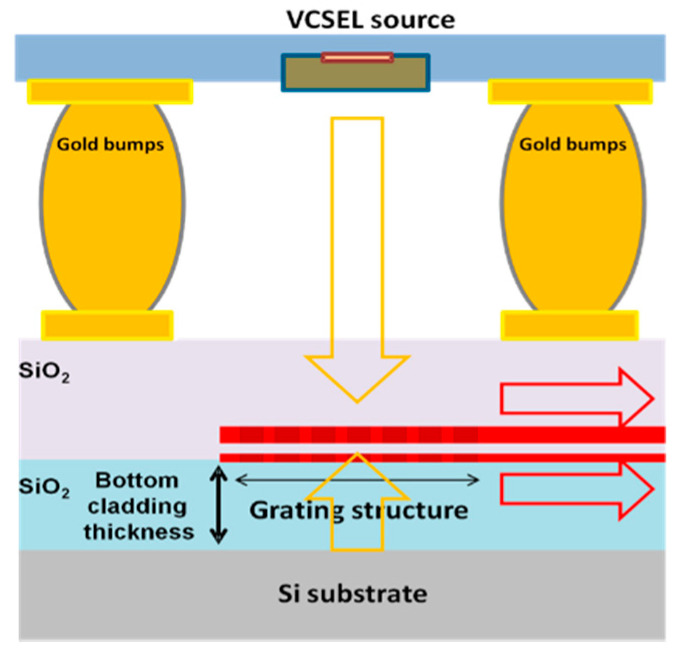
Basic grating coupler working principle.

Moving to the integration of the platforms towards a compact photonic sensor device, a novel design of a photonic platform has been developed by combining the above-described photonic chip with VCSEL, photodiodes and grating couplers. In those platforms, the light is coupled in and out of the chip by means of a grating structure instead of butt-end coupling (Figure 8).

**Figure 9:**
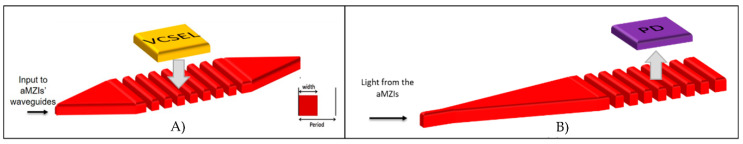
(**A**) “two-port” and (**B**) “single port” grating coupler for in and out coupling of the light, respectively.

In-coupling of light is achieved by using a two-port grating coupler [26] (Figure 9A in order to trap the light from a VCSEL that is placed on top of the grating structure, while out-coupling of the light from each aMZI sensor output waveguide, towards a photodiode, is realized by means of a single port grating coupler [27] (Figure 9B).

**Figure 10:**
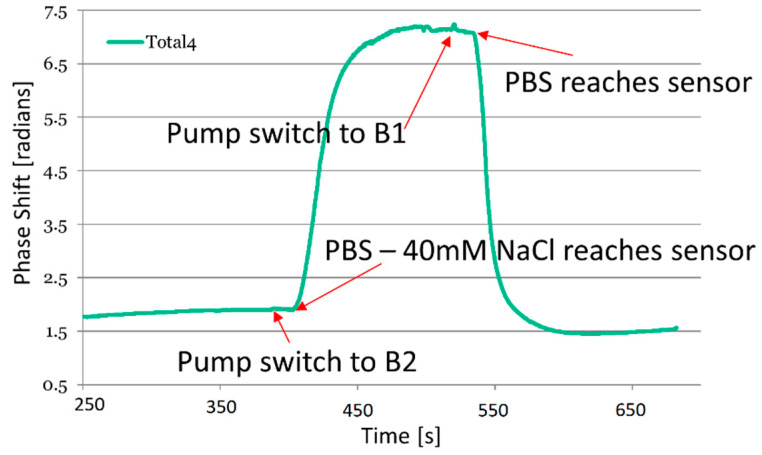
Refractive index shift (in radians) during the alternating flow sequence of PBS and PBS with reduced NaCl concentration (−40 mM). The results were recorded with the integrated photonic biosensor device for cancer treatment monitoring.

The functionality of the integrated photonic biosensor device was tested by measuring the bulk RI changes of two alternating buffer solutions (Figure 10): Phosphate buffered saline pH 7.2 (PBS) and PBS with NaCl concentration reduced by 40 mM. During each alternating cycle, 60 μL of liquid passes through the sensor with a flow rate of 48 μL/min pumped by one of the syringes at a time. This flow rate allows for the concentration profiles to approach stable level in each phase.

Figure 10 depicts the refractive index changes as recorded with the integrated sensor device. The priming of the complete system was performed within the first 250 s to make sure no air was left in the microfluidic channels towards the photonic platform and on the platform itself. Syringe B1 is filled with PBS while syringe B2 is filled with PBS with a reduced NaCl concentration. During B1 pumping, a drift can be seen from 250 s to the pumping syringes being switched to B2 attributed to temperature fluctuations. As indicated by the arrows in Figure 10, a disturbance due to the pumps being switched can be observed, and after about 15 s, the signal on the photonic platform starts to change significantly. The concentration change reaches a stable level before the liquids are changed.

## 4. Conclusions

In this paper, we present the development of a miniaturized, reliable biosensing system for accurate and highly sensitive detection of protein cancer biomarkers, for the treatment monitoring of cancer disease and the therapeutics response. This device relies on the integration of different platforms (photonic, bioassay, functionalization, disposable microfluidics cartridge) to one compact instrument. The validation of the device was performed by two approaches. The photonic platform was validated in the presence of spiked samples with increasing complexity and real blood serum samples from a breast cancer patient. The integrated device with the disposable microfluidic platform and the photonic platform was tested for the detection of bulk RI measurements with alternating standard PBS buffer and PBS buffer with reduced NaCl concentration.

## Data Availability

Not applicable.
